# Supplementary figures and images for: Identification of four hub genes associated with adrenocortical carcinoma progression by WGCNA
Source: PeerJ. 2019 Mar 14;7:e6555. doi: 10.7717/peerj.6555 (PMC6421058; doi:10.7717/peerj.6555)

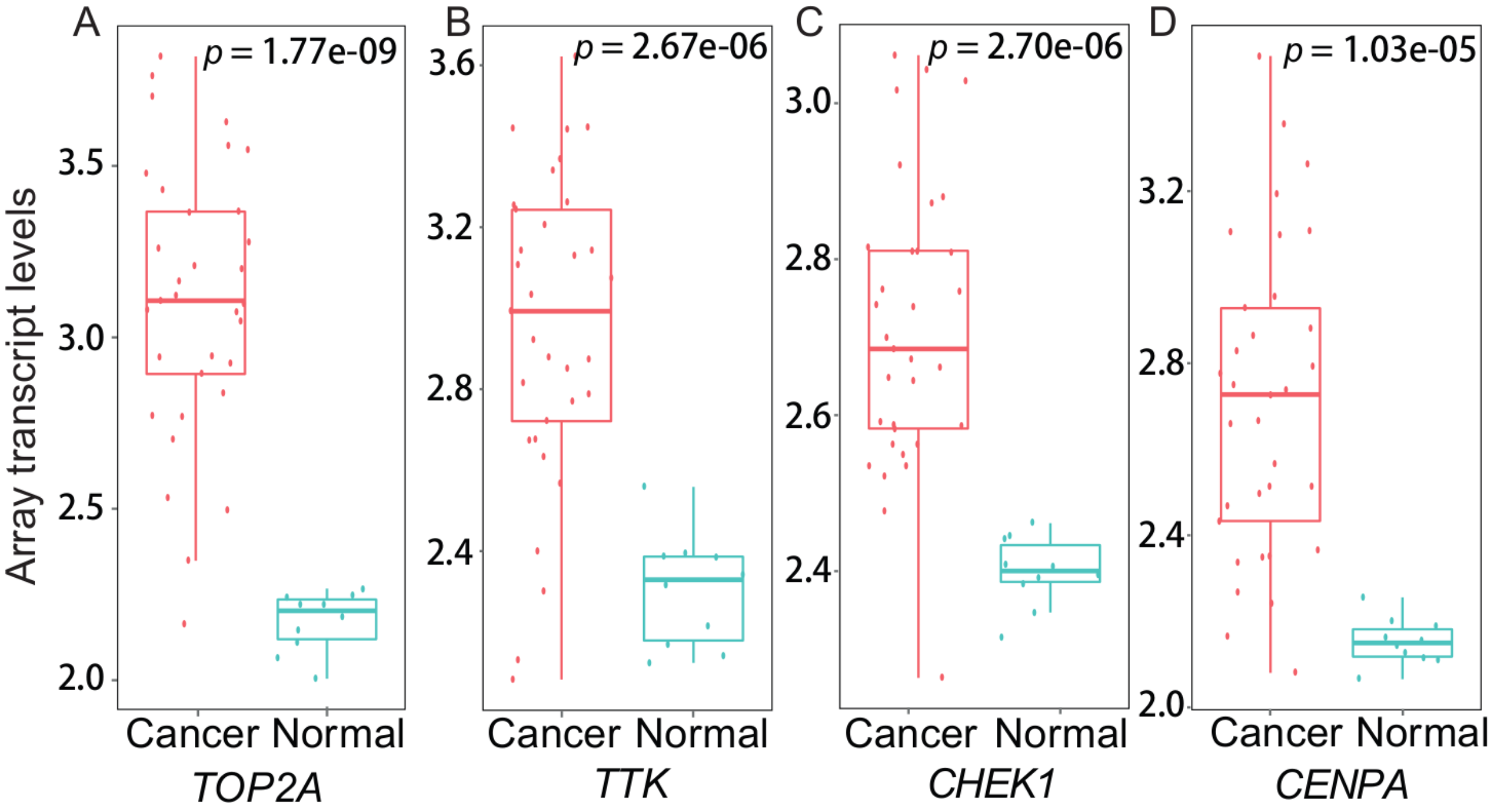

Supplement: Supplemental Information 1 — (A-D) The four hub genes significantly expressed in ACC samples compared with normal tissue samples in GSE10927 dataset. [file peerj-07-6555-s001.png]
